# Colitis reduces active social engagement in mice and is ameliorated by supplementation with human microbiota members

**DOI:** 10.1038/s41467-024-46733-7

**Published:** 2024-03-30

**Authors:** D. Garrett Brown, Michaela Murphy, Roberto Cadeddu, Rickesha Bell, Allison Weis, Tyson Chiaro, Kendra Klag, Jubel Morgan, Hilary Coon, W. Zac Stephens, Marco Bortolato, June L. Round

**Affiliations:** 1 Department of Pathology, University of Utah School of Medicine, Huntsman Cancer Institute, Division of Microbiology and Immunology, Salt Lake City, UT USA; 2 Department of Pharmacology and Toxicology University of Utah College of Pharmacy, Salt Lake City, UT USA; 3 Department of Psychiatry, University of Utah School of Medicine, Salt Lake City, UT USA

**Keywords:** Applied microbiology, Diseases of the nervous system

## Abstract

Multiple neurological disorders are associated with gastrointestinal (GI) symptoms, including autism spectrum disorder (ASD). However, it is unclear whether GI distress itself can modify aspects of behavior. Here, we show that mice that experience repeated colitis have impaired active social engagement, as measured by interactions with a foreign mouse, even though signs of colitis were no longer present. We then tested the hypothesis that individuals with ASD harbor a microbiota that might differentially influence GI health by performing microbiota transplantation studies into male germfree animals, followed by induction of colitis. Animals that harbor a microbiota from ASD individuals have worsened gut phenotypes when compared to animals colonized with microbiotas from familial neurotypical (NT) controls. We identify the enrichment of *Blautia* species in all familial NT controls and observe an association between elevated abundance of *Bacteroides uniformis* and reductions in intestinal injury. Oral treatment with either of these microbes reduces colon injury in mice. Finally, provision of a *Blautia isolate* from a NT control ameliorates gut injury-associated active social engagement in mice. Collectively, our data demonstrate that past intestinal distress is associated with changes in active social behavior in mice that can be ameliorated by supplementation of members of the human microbiota.

## Abstract (web summary)

Past intestinal distress is associated with diminished social behavior in mice. Here, the authors show that treatment with microbiota members that are enriched in neurotypical people versus people with ASD can ameliorate colitis severity and associated sociability deficits.

## Introduction

Multiple neurological disorders including ASD, MS, and PD are known to be influenced by a combination of genetic, neurobiological, immunological and environmental factors. More recently, one of the environmental factors found to be involved in these diseases is the microbiota^1–5^. Many individuals that have these diseases are comorbid for GI symptoms such as diarrhea, constipation, abdominal pain, reflux and bloating which often correlates with the severity of neurological deficits^6–8^. Other GI dysfunctions, including reduced GI motility and increased gut permeability, have also been reported^9,10^. In addition, a large multicenter trial of over 14,000 individuals reported a higher incidence of inflammatory bowel disease (IBD) in individuals with ASD^11^. These data underscore the notion that intestinal factors, including the gut microbiota, might modify the presentation of neurological disease manifestations such as behavior.

Several studies have identified differences in the microbiota between ASD-affected individuals and neurotypical (NT) controls (individuals without neurological or psychiatric diagnoses), prompting further investigations into the modulatory effects of gut bacteria on behavior^12–17^. Indeed, commensal bacteria have been shown to affect many complex behaviors in several animal models^18,19^. In an open-label study, individuals with ASD that received a fecal microbiota transplant (FMT) from a NT donor had significant improvements in behavior and GI symptoms^20^. Moreover, transplantation of the microbiota from individuals with ASD, MS or PD intro mice led to modifications of the respective disease^2–4^. Thus, there is evidence in animal models and human trials that the gut microbiota can modulate behavioral outcomes in a variety of neurological diseases. However, since many of these studies utilize genetic modifications associated with that particular disease, it is difficult to disentangle the effects of the gene, microbiota and/or intestinal inflammation on the behavioral outcome. In addition, though FMT is currently being trialed as a therapy in several diseases, the complexity of an FMT makes it difficult to obtain reproducible donor microbiotas. There is also the possibility of transferring pathobionts into the recipient. Thus, the identification of specific microbiota members that can ameliorate GI symptoms and modify neurological manifestations will be important for future management of these disorders^21–23^.

Here, we employ a model of repeated intestinal injury and microbiota transplantation to test the hypothesis that the microbiota harbored by individuals with a neuro-developmental disorder might directly influence GI distress, which could modify subsequent behavioral outcomes. We show that past intestinal distress is associated with changes in active social behavior in mice, as they spend less time interacting with an unfamiliar (here defined as an animal that the test mouse has not been exposed to prior to the experiment) mouse than mice naive to intestinal insult. We also demonstrate that transplantation of the microbiota from individuals with ASD into mice is sufficient to induce worsened gut phenotypes upon colitis induction compared to mice colonized with microbiotas from familial NT controls. Since we are interested in understanding the specific microbiota members that contribute to these phenotypes, we analyzed the microbiota compositions. There is an enrichment of *Blautia* species in all familial NT controls and association between *Bacteroides uniformis* abundance and reduced intestinal injury in mice. We also show that oral treatment with *B. uniformis* or a NT control *Blautia* isolate reduces colitis severity and that provision of the *Blautia isolate* ameliorates colitis-associated sociability in mice.

## Results

### Animals that experience repeated colitis have decreased active social interactions

While there appears to be a clear connection between gut dysfunction and diseases of the CNS, it is unclear to what degree intestinal ailments themselves contribute to behavioral abnormalities. A few studies have begun to test this in acute models of dextran sulfate sodium (DSS)-induced colitis. In these studies, animals exhibited increased anxiety-like behaviors and memory loss during the active phase of disease. These behavioral deficits were often reversed when the damaging agent was discontinued and the disease resolved^24–27^. As animals actively experiencing intestinal injury develop diarrhea, stop grooming, become hunched and lose significant amounts of weight, it is perhaps not surprising that they also have behavioral differences. However, most individuals with comorbid GI disease and behavioral deficits experience frequent bouts of intestinal dysfunction whereby GI symptoms can relapse and remit but behavioral symptoms remain more constant. One study compared an acute and repeated model of DSS treatment and observed fewer behavioral abnormalities in animals having repeated disease^28^. However, behavioral responses were still analyzed just one day after the last DSS treatment, when animals were still actively experiencing disease. Thus, further experiments are needed to understand whether repeated intestinal complications can modify behavioral outcomes in mice during the non-active phase.

**Fig. 1:**
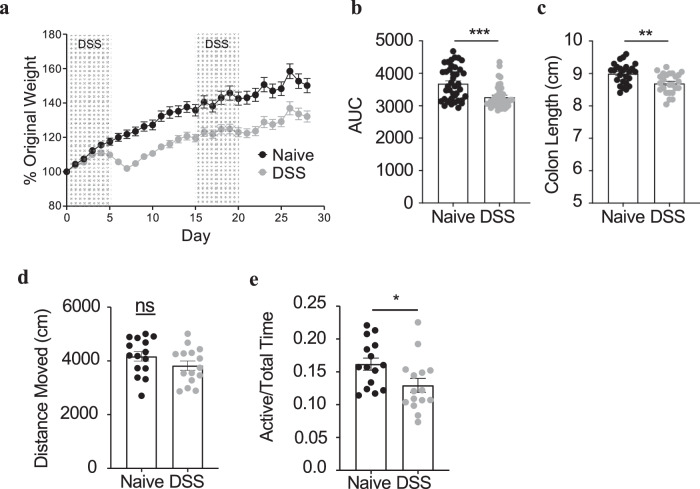
Repeated DSS injury induces social deficits in mice. **a** Percent of original weight of 3–4-week-old male mice given DSS (gray) or control mice (black). Background shading illustrates treatment with DSS. (**b**) Area under the curve (AUC) of the data represented in (**a**), (*P* = <0.0001). At the end of behavioral testing, day 51, mice were weighed, and euthanized. Colon length (**c**) was assayed (*P* = 0.0017). **a**–**c** *n* = 40 animals/treatment analyzed over 4 independent experiments. At day 30 of schedule, mice were analyzed for behavioral changes. **d** Total movement in the open-field test (*n* = 15/treatment). **e** Proportion of total time spent actively socializing with an unfamiliar mouse (*n* = 15/treatment, *P* = 0.0286). Statistics: All bars/lines represent mean values +/− standard error of the mean (SEM). ns *P* > 0.05, *P < 0.05, **P < 0.01, ***P < 0.001 for two-tailed unpaired Student’s *T* test (**b**–**e**).

To complement these published studies, we treated mice with a 30-day time course of repeated DSS, consisting of 5 days of 2.5–3.0% DSS treatment, 10 days of regular water, followed by another 5 days of DSS and ten days of water. This model allows for control of the timing of intestinal damage and permits periods when the intestines can repair and animals can regain weight lost during the DSS treatment^29^. This model is also well known to increase gut barrier permeability, a feature associated with several neurological disorders, including ASD. At the end of the 30 days, we performed a battery of behavioral tests to measure anxiety- and depression-related responses as well as compulsivity and sociability. Consistent with appropriate induction of disease, DSS-treated mice weighed significantly less over the entire course of observation (Fig. 1a, b and Supplementary Fig. 1a, b), displayed reduced colon length (Fig. 1c), and had elevated fecal lipocalin-2 levels (Supplementary Fig. 1c). Importantly, however, animals that experienced repeated GI injury did not exhibit significant differences in locomotor activity (Fig. 1d) or apparent signs of sickness, as they were appropriately groomed, had no diarrhea, and did not display a hunched posture or other general signs of disease such as inflammation of the eyes or swelling of the face as would be seen in the acute DSS injury model.

In contrast to published findings, during acute GI insult, we observed no differences in anxiety-like behavior as measured by the elevated plus-maze and open-field tests (Supplementary Fig. 1d–f). Moreover, we did not observe differences in compulsive or depressive-like responses as measured by the marble burying and the tail suspension tests, respectively (Supplementary Fig. 1g–j). These results suggest that repeated GI disease does not globally impact behavior and that anxiety-like behavior might only be associated with acute inflammation. To assess alterations in sociability, we conducted the three-chamber test. Both groups of mice entered the social chamber with the foreign mouse more often than the control chamber (Supplementary Fig. 1k). However, animals that had experienced repeated GI insult spent less time actively sniffing and interacting with the foreign caged mouse (Fig. 1e), demonstrating that animals with intestinal injury display deficits in their propensity to engage in social interactions with novel mice. Overall, these data suggest that past, repeated GI insults are associated with abnormal social interactions in mice.

### Microbiota transplantation from individuals with ASD alters intestinal phenotypes in mice

ASD is one of multiple neurological disorders with observed gastrointestinal comorbidities, and depending on the study, up to 85% of individuals with ASD also have GI complaints^6^. While one group has demonstrated that transplantation of the microbiota from individuals with ASD can alter behavior in mice, the impact of the microbiota from individuals with ASD on intestinal health was not tested^3^. Our data suggest that repeated GI insults are associated with a decreased propensity to engage socially, a characteristic feature of ASD. Thus, some individuals with ASD might harbor a microbiota that reduces intestinal health and subsequently modifies behavioral phenotypes. Based on this, we sought to determine whether the microbiota from individuals with ASD could alter disease severity induced by an acute course of DSS treatment. We chose this model to screen several microbiotas for alterations in disease severity because it is reproducible, quick and provides clear outcomes that are easy to assess in a large number of animals^29^.

**Fig. 2:**
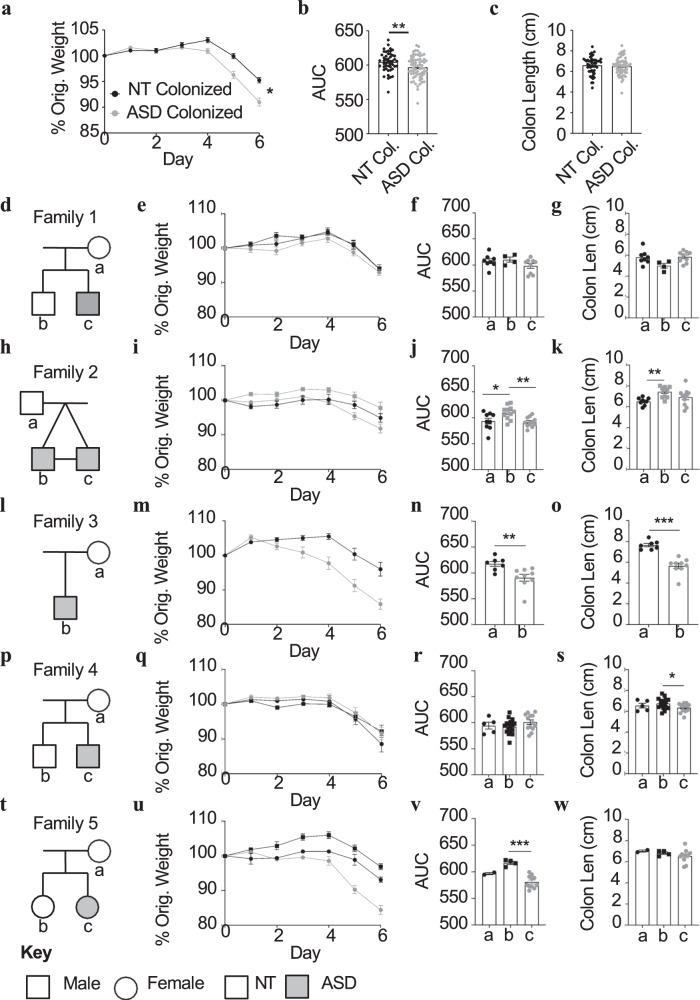
DSS-induced intestinal phenotypes are worsened by the microbiota of individuals with ASD. In all, 6–8-week-old male offspring of animals colonized with human microbiotas were subjected to DSS treatment for 7 days (*n* = 2–15 animals/microbiota). **a** Percent of original weight in NT-colonized (black) or ASD-colonized (gray) mice. **b** Area under the curve (AUC) of percent of original weight as shown in (**a**) (*P* = 0.0038). **c** Colon length at day 7 of DSS injury. **d**, **h**, **l**, **p**, **t** Pedigrees of five families included in the study. Gray shading represents ASD and white represents neurotypical controls. Circle indicates a female and square indicates a male. **e**, **i**, **m**, **q**, **u** Percent of original weight in NT-colonized (black) or ASD-colonized (gray) mice. **f**, **j**, **n**, **r**, **v** AUCs associated with the curves in (**e**, **i**, **m**, **q**, **u**). **g**, **k**, **o**, **s**, **w** Colon lengths at the end of DSS treatment time course. Statistics: *P < 0.05, **P < 0.01, ***P < 0.001 for two-tailed unpaired Student’s *T* test (**b**, **n**, **o**), and one-way ANOVA with Tukey’s multiple comparisons (**j**, **k**, **s**, **v**).

Fecal samples were collected from households containing at least one member with ASD, and other household family members were used as neurotypical (NT) controls. Use of household, familial samples better controls for some environmental, genetic, and dietary differences that may also drive alterations in the microbiota^30,31^. Five donor families were identified through the Utah Population Database (Supplementary Table S1). ASD donors consisted of four males and one female, while household control donors consisted of four females and four males. The household controls were either unaffected sibling controls or parents of the individuals with ASD (Fig. 2 and Supplementary Table S1).

As in previous studies, donor microbiota samples were transplanted into germ-free breeder animals and the offspring of these “humanized” breeders (referred to as H-F1 offspring) were provided DSS for 7 days^3^ (Supplementary Fig. 2a). In this model, weight loss and colon shortening are two commonly analyzed parameters used for gross examination of wasting and intestinal damage. When all H-F1 animals receiving NT microbiotas or microbiotas from individuals with ASD are grouped together, there are no significant differences in colon shortening; however, animals receiving the microbiota from ASD individuals lost more weight when compared to animals receiving a microbiota from a neurotypical donor (Fig. 2a–c). Yet, when each microbiota is treated as a single sample for weight loss, the effect size of weight loss in mice given a microbiota from patients with ASD is reduced (*P* = 0.0627) (Supplementary Fig. 2b).

Analysis of the data when grouped by family revealed that the microbiota from all but one individual with ASD consistently worsens either weight loss, colon shortening or both when compared to the respective familial controls (Fig. 2d–w). Animals colonized with the ASD microbiota from families 2–5 lost significantly more weight and/or had significantly shorter colons when compared to at least one of their household NT controls (Fig. 2h–w). Family 2 has a household control that has been diagnosed with ulcerative colitis, and indeed, it appears this microbiota leads to similar weight loss as one of the individuals with ASD (Fig. 2h–k). Within family 3, the microbiota from the individual with ASD led to significant weight loss and colon shortening compared to the neurotypical mother (Fig. 2l–o). In family 4, mice colonized with the microbiota of the ASD donor had a significant decrease in colon length when compared to a same-sex NT sibling, but not the parent (Fig. 2p–s). Family 5 contains a sex-matched sibling control without ASD and transplantation of this microbiota does not lead to as much weight loss as transplantation of the microbiota from the individual with ASD (Fig. 2t–w). Thus, in most cases, microbiotas from individuals with ASD worsen GI symptoms. Collectively, these data support that the microbiota possessed by some individuals with ASD can exacerbate GI insult.

### Identification of human gut microbiota members that reduce DSS injury

**Fig. 3:**
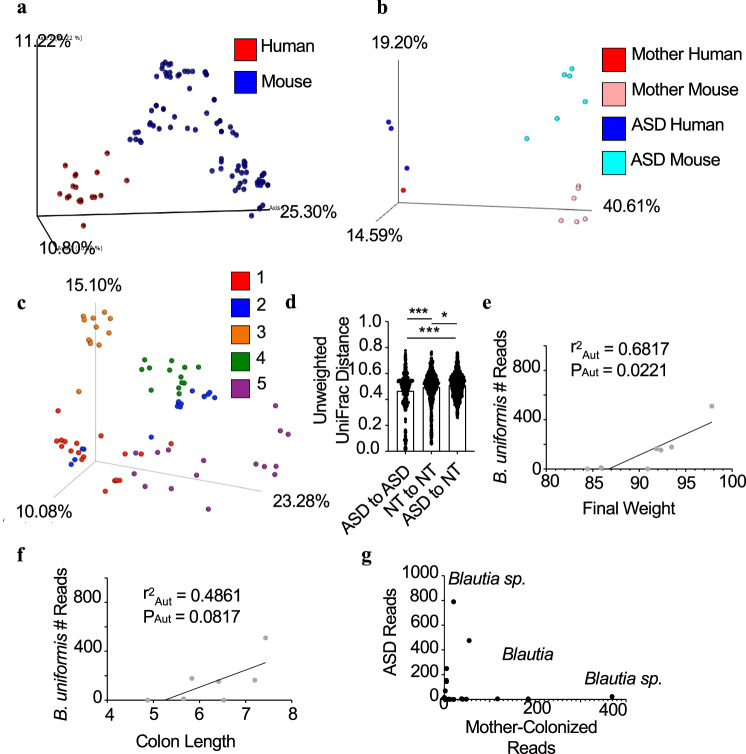
Identification of microbes that influence intestinal injury severity. **a** PCoA plots of Unweighted Unifrac distances, colored by source: red = human, blue = mouse. **b** PCoA plot of Unweighted Unifrac distances in family 3, colored by source and donor: red = human mother microbiota, pink = mouse with mother microbiota, blue = human with ASD microbiota, and cyan = mouse with ASD microbiota (**c**) PCoA plot of Unweighted Unifrac distances of colonized mice, colored by family: red = family 1, blue = 2, orange = 3, green = 4, purple = 5. **d** Unweighted UniFrac distance values comparing within-donor neurotype and between-donor neurotype variation (*n* = 528–1419/comparison). Bars represent mean values +/− SEM. **e** Regression of mean *B. uniformis* number of reads with mean final percent of original weight in ASD and NT-colonized mice. **f** Regression of mean *B. uniformis* number of reads with endpoint colon length. Coefficient of determination (*r*^2^) = 0.6817 and *P* = 0.0221. **g** Taxa significantly enriched (by ANCOM) in either ASD or NT mother in family 3, with *Blautia* sp. labeled. Statistics: *P < 0.05, ***P < 0.001 for one-way ANOVA with Tukey’s multiple comparison (**d**).

Donor input samples and large intestinal contents from 6 to 8-week-old offspring were subjected to 16S sequencing to interrogate their microbiotas. In this case, the source of the sample (sequenced inputs of human feces or output from *F1* progeny of colonized mice) strongly dictated community similarity and clustered together (Fig. 3a, b). To characterize the microbiota structure in colonized mice, we excluded the human input samples and measured unweighted UniFrac distances between mice colonized with the same microbiota compared to mice colonized with different microbiotas. UniFrac distances are smaller within microbiotas than between microbiotas (Supplementary Fig. 3a). When analyzing microbiotas from an individual family and including human donors, PCoA plots show distinct clustering by source and microbiota (Fig. 3b). The plot’s vertical axis (PCoA axis 2; explaining 19.20% of variation) separates samples by microbiota and not by source. Taken together, the microbiotas of the recipient animals cluster together and more closely resemble the community of the donor sample than that of other animals transplanted with samples from different donors. When analyzing colonized mice via unweighted UniFrac, PCoA plots suggested that microbiota composition seemed to cluster by donor family (Fig. 3c). By comparing UniFrac values within and between each family, we found that the mean distance within each family is significantly less than between each other family (Supplementary Fig. 3b–f). These data suggest that donor family strongly dictates community structure and support that transplantation of human microbiotas to animals maintains features of the human donor.

Several reports have identified distinctions between microbiotas from ASD and NT individuals^3,16,17^. Transplantation of the microbiota from an individual with ASD into mice can worsen some behavioral outcomes suggesting that the microbiota from an individual with ASD influences behavior abnormalities^3^. Moreover, fecal microbiota transplantation (FMT) from NT into ASD patients is associated with a reduction in severity of behavioral abnormalities^20^. As microbes can influence GI function and GI function may influence CNS function, we were interested in identifying specific members of the microbiota that correlated with intestinal phenotypes. Initially, animals were analyzed to determine if neurotype influenced community composition. By comparing unweighted UniFrac distances, we found that donor neurotype also predicts microbiota structure; within-neurotype distance is smaller than between-neurotype distance (Fig. 3d). There was also more distance within our NT-colonized mice than our ASD-colonized mice. Like previous reports from humans with ASD, we found that mice colonized with ASD microbiotas had less Actinobacteria and Firmicutes and more Proteobacteria (Supplementary Fig. 3g). Interestingly, when comparing individual taxa that were differentially abundant between neurotypes, only one was significantly different. This amplicon sequence variant (ASV) was a *Eubacterium sp*. that was only detected in animals colonized from ASD donors (Supplementary Fig. 3h). While we found this microbe in four of the seven cohorts of mice colonized with the ASD microbiotas, we did not detect it in any NT-colonized mice. This finding agrees with a recent report that individuals with ASD had high *Eubacterium* prior to receiving an FMT^32^.

We performed a linear regression between mean abundance of individual taxa and weight or colon length of all transplanted animals. From these regressions, we identified multiple ASVs, including an Enterobacteriaceae, that were negatively associated with final weight (Supplementary Fig. 3i) and thus were increased in abundance in animals that lost the most weight during DSS treatment. When looking at ASD-colonized mice, the only organism that was identified to be positively associated with final weight and a trending relationship with colon length was *Bacteroides uniformis* (Fig. 3e, f). This data suggested that *B. uniformis* might be associated with protection from intestinal injury in ASD-colonized mice. In the literature, the presence of *B. uniformis* in healthy human volunteers distinguishes them from individuals with irritable bowel syndrome and ulcerative colitis, and individuals with Crohn’s disease have a lower abundance of *B. uniformis*, supporting our finding of the association of *B. uniformis* with better intestinal disease outcomes^33,34^.

The human microbiota is known to have high interpersonal variability, and it is likely that many microbiota-influenced diseases are not a result of a gain or loss of a single universal organism. Based on this, we wondered if we could identify organisms of interest by taking a more personalized analysis of the microbiota from a single family. Initially, we focused on the microbial differences in family 3 as the intestinal disease severity was most distinct between the NT control and ASD-colonized mice (Fig. 2l–o). Sequencing of the two microbiotas from this family suggested that distinct members of the genus *Blautia* were differentially abundant between the NT control and ASD-colonized animals (Fig. 3g). Interestingly, *Blautia* species have been reported to be significantly reduced in individuals with ASD that are also comorbid for GI disease^35^. We analyzed other families to determine whether there are differences in *Blautia*. When regressing *Blautia sp*. frequency against weight loss, we do not observe a significant relationship (Supplementary Fig. 3j). However, when analyzing the microbiotas from families individually, there is a significant difference in *Blautia* species across multiple families. Indeed, in all families, ASD-colonized mice have less *Blautia sp*. than either a sibling (families 2 and 3) or parental (families 1, 4, and 5) control (Supplementary Fig. 3k). Thus, personalized analysis in animals transplanted with human microbiota can reveal organisms of interest. Overall, our transplantation studies have highlighted two potential candidate bacterial organisms that are significantly correlated with worsened intestinal outcomes in mice and appear to be reduced in individuals with ASD and GI problems, suggesting that perhaps these organisms can reduce intestinal injury or disease.

### Oral treatment with *B. uniformis* reduces intestinal disease in mice

**Fig. 4:**
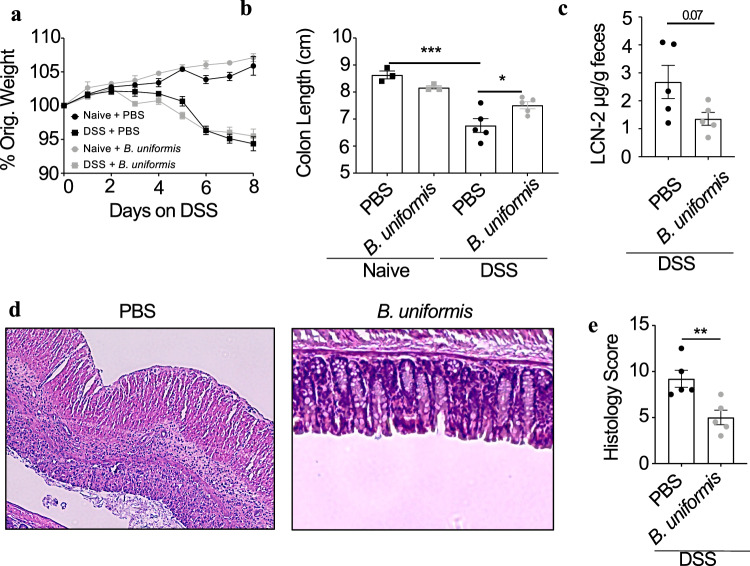
*Bacteroides uniformis* can reduce DSS severity. In all, 6–8-week-old male SPF mice (*n* = 5) were gavaged with *B. uniformis* or PBS and then subjected to DSS treatment. Squares represent DSS-treated mice, circles represent controls. Black represents *B. uniformis-*treated mice, gray represents PBS-treated controls. **a** Percent of original weight in mice. **b** Colon length and **c** fecal lipocalin (*P* = 0.0711) at the end of DSS treatment. **d** Representative histology images and **e** associated scores from DSS-treated mice (*P* = 0.0088). Statistics: all bars/lines represent mean values +/- SEM. *P < 0.05, **P < 0.01, ***P < 0.001 for one-way ANOVA with Tukey’s multiple comparison (**b**) and two-tailed unpaired Student’s *T* test (**c**, **e**).

To determine how *B. uniformis* modifies intestinal injury, we orally gavaged animals with 10^7^ CFU/day of *B. uniformis* 2 weeks prior to initiation of acute DSS injury. *B. uniformis* had little effect on animals in the absence of DSS treatment, suggesting that oral gavage of this bacteria is well tolerated; however, while *B. uniformis-*treated animals lost a similar amount of weight compared to mock-treated animals during DSS injury, *B. uniformis-*treated animals had significantly longer colons and less fecal lipocalin-2 (Fig. 4a–c). Blinded histological analysis also showed that *B.* uniformis-treated mice had similar immune cell infiltrate into the tissue yet had significantly decreased crypt loss (Fig. 4d, e and Supplementary Fig. 4). Based on this data, the increased abundance of *B. uniformis* identified in NT individuals would be predicted to protect from intestinal injury and inflammation.

### *Blautia sp*. from a NT individual reduces intestinal injury in humanized mice

**Fig. 5:**
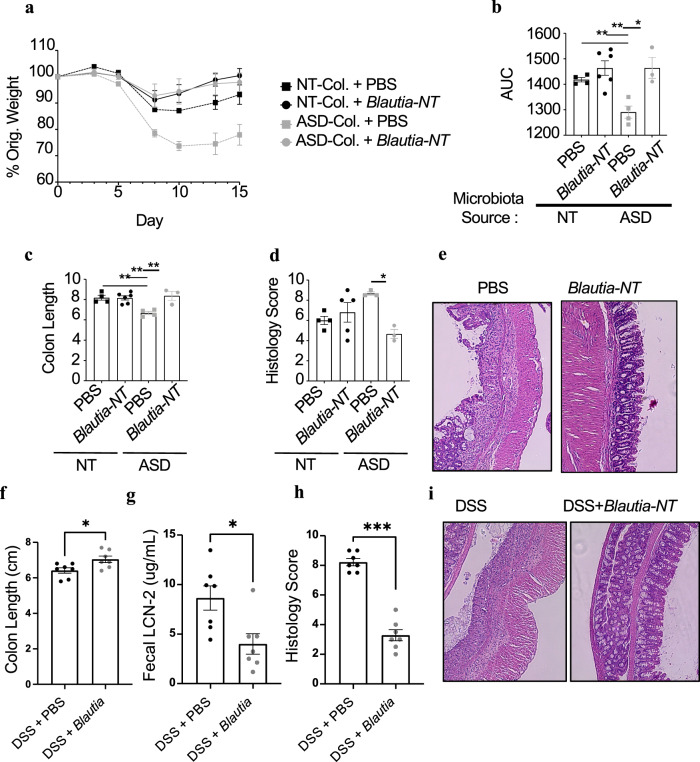
*Blautia-*NT isolated from a NT individual reduces colitis severity. Mice colonized with the microbiota from family 3 were given DSS and PBS or *Blautia-*NT supplementation. Circles represent *Blautia-*treated mice, squares represent controls. Black represents NT-colonized mice, gray represents ASD-colonized mice. **a** Percent of original weight loss. **b** AUCs associated with (**a**). **c** Colon length at the end of DSS colitis. **d** Histology scores (*P* = 0.0238) and **e** representative images at the end of DSS colitis. **a**–**d** *n* = 3–6 animals/treatment. **f–i** SPF mice that did or did not receive *Blautia-*NT treatment before and during acute DSS (7 days of 2.5%). Black circles represent PBS-treated mice and gray circles represent *Blautia-*NT treated mice. **f** Colon length (*P* = 0.0268), **g** fecal lipocalin-2 (*P* = 0.0111), **h** histology scoring (*P* = 0.0006), and **i** representative histological images. **f**–**i** *n* = 7 4-week-old animals/treatment. Statistics: all bars/lines represent mean values +/− SEM. *P < 0.05, **P < 0.01, ***P <  0.001 for two-way ANOVA with Tukey’s multiple comparison (**b**, **c**) and Mann–Whitney test (**f**–**h**).

Our analysis of family 3 revealed distinct differences in *Blautia* species between the NT and the ASD individuals. Therefore, we assembled a culture collection of microbes from NT-colonized mice in order to isolate *Blautia* species that were associated with reduced intestinal disease. We cultured multiple isolates of *Blautia* and identified one with 99% 16 S rDNA similarity to the *Blautia* overrepresented in the maternal-NT sample (now referred to as *Blautia-*NT with isolate name: JLR.GB024). To test *Blautia*-NT’s influence on disease severity, we colonized germ-free mice with the original family 3 ASD and NT microbiotas. Two weeks before DSS, a subset of these animals were gavaged with 10^7^ CFU/day of *Blautia*-NT. Mice colonized with the ASD microbiota again lost significantly more weight and had shorter colons than the NT-colonized mice (Fig. 5a–c). In addition, we performed histological analysis on these animals and identified that animals colonized with the ASD microbiota had significantly higher disease scores that indicated greater intestinal injury (Fig. 5d, e and Supplementary Fig. 5). However, oral treatment of ASD-colonized animals with *Blautia-*NT significantly protected animals from weight loss, colon shortening and crypt loss (Fig. 5a–e). The *Blautia-*NT treatment did not affect disease in mice colonized with the NT microbiota. We speculate that the lack of effect might be due to either the already high levels of *Blautia* in the colonized mice or the already limited disease conferred by this microbiota. To test this, we orally gavaged *Blautia*-NT into wild-type specific pathogen-free (SPF) mice prior to induction of DSS colitis. Our SPF mouse colony lacks this *Blautia* strain and possesses a microbiota that leads to moderate colitis (Fig. 4). Oral treatment with *Blautia-*NT into SPF mice also potently protected from colitis severity. Indeed, animals treated with *Blautia*-NT had longer colons, reduced lipocalin-2 levels and reduced intestinal damage (Fig. 5f–i). These data demonstrate that *Blautia*-NT can reduce intestinal disease in multiple microbiota backgrounds that are conducive to moderate colitis and lack *Blautia* sp.

### *Blautia-*NT ameliorates intestinal injury-associated asocial behavior

**Fig. 6:**
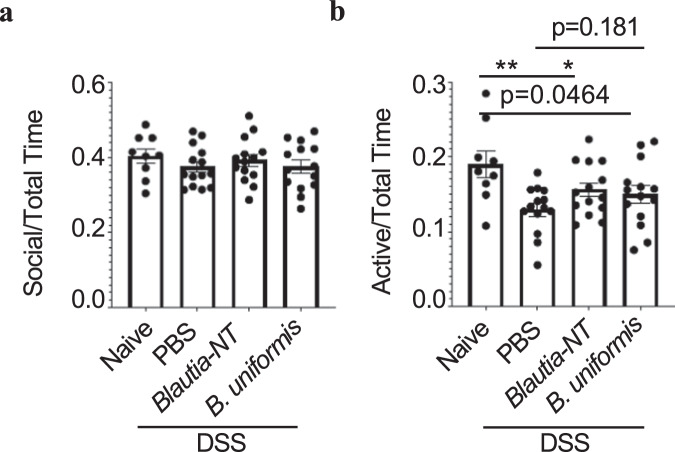
*Blautia-*NT treatment ameliorates intestinal injury-associated defects in social interactions. Mice undergoing a repeated DSS course were treated with either *Blautia-*NT*, B. uniformis* or PBS and subject to three-chamber testing (*n* = 9 or 14/treatment). **a** Ratio of time spent socializing to total time. **b** Ratio of total active time engaging with an unfamiliar mouse to time spent in social chamber. Statistics: all bars represent mean values +/− SEM. *P < 0.05, **P < 0.01 for unpaired, two-tailed *T* test.

Our data demonstrate that repeated intestinal injury is associated with reduced social interactions in mice. As we have identified two organisms from the human microbiota that reduce DSS-induced intestinal injury, we wondered if these organisms could be used to ameliorate intestinal injury-induced reductions in social behavior. To test this, we performed the three-chamber test after treating animals orally with either *Blautia*-NT or *B. uniformis* during repeated, intermittent DSS insult. Again, animals that had experienced repeated intestinal damage had no differences in locomotion but were significantly less interactive with the unfamiliar mouse when compared to age and sex-matched treatment-naive mice (Fig. 6a, b). However, while mice orally gavaged with *B. uniformis* had no significant changes (*P* = 0.181) in sociability, animals treated with *Blautia-*NT displayed significantly increased social engagement with unfamiliar animals compared to animals that had experienced intestinal insult (Fig. 6b). Collectively, our data demonstrate that intestinal injury alone can influence certain aspects of behavior and that some individuals with ASD might lack members of the microbiota that can protect or improve intestinal complications and behavioral outcomes.

## Discussion

A recent literature review analyzed the data from all human studies involving GI symptoms in ASD and identified that over 70% of these studies reported statistically significant differences in the occurrence of GI complications in individuals with ASD, confirming common reports of the prevalence of intestinal abnormalities in this behavioral disorder^35^. Similar observations have been made for other neurological diseases, including PD and MS^21,22^. All these ailments involve complex interactions between genetics, environment, diet and the immune and nervous systems; thus, understanding what components of these diseases influence behavioral symptoms becomes difficult to disentangle. The use of mouse models can help to tease apart these differences. Several investigations have used genetic modes whereby animals possess polymorphisms or deletions in genes known to be associated with the disease^2,4,36^. However, in these models, the genetic deletions are often not specific to the brain and could also influence the developing gut and microbiota. Thus, a major question left unanswered is whether microbiota-driven GI complications alone can influence behavioral abnormalities.

On this basis, we reasoned that a model of repeated gut injury would allow us to definitively test this. The use of the DSS model seemed ideal for this purpose because it allows for specific timing of gut injury and periods of recovery prior to behavioral testing. This is an essential distinction from the few studies that have looked at behavior in the acute DSS model. In this model, animals are actively losing weight, have stark diarrhea and often appear moribund which would be expected to manifest in behavioral differences due to sickness^37^. In addition, this model can be performed in wild-type animals and, therefore, is not complicated by genetic deficiencies or early life perturbations that may also indirectly influence gut or brain activity. Using this model, we observed that while animals that experienced repeated gut injury entered the social chamber as often as non-DSS mice, they displayed decreased active social engagement in the form of sniffing, pawing, and interacting with an unfamiliar mouse. This demonstrates that intestinal distress might modify the way an individual engages socially with others. The reason why intestinal distress modifies social engagement is unclear and is an active area of research. It is possible that despite having the appearance of health, the animals might still be experiencing visceral pain that could modify their behavior. Future work in this area could address this possibility. Our results suggest GI distress in individuals with neurological diseases, such as ASD, might modify their behavioral symptoms and that treating gut complications could possibly ameliorate some behavioral abnormalities seen in neurological diseases.

Given the wealth of literature demonstrating the direct effects of the gut microbiota on intestinal wound repair, inflammation and severity of diseases like IBD, we tested whether the microbiota harbored by individuals with ASD can influence the severity of GI insult^38^. One study that transplanted ASD microbiotas into naive germ-free animals observed no differences in one parameter of GI health, intestinal permeability, based on microbiota composition alone^3^. However, modeling intestinal manifestations in mice requires a variety of factors. Indeed, in studies testing the role of the microbiota in IBD induction, transplantation of the microbiota from IBD patients alone is not sufficient to elicit disease in mice but requires an additional genetic or chemical induction factor^39^. Thus, we again used DSS to test the potential of the microbiota harbored by individuals with ASD to influence intestinal disease severity. Of note, we did survey the individuals from this study for GI complaints. Consistent with the literature, most individuals with ASD self-reported some sort of GI complaint. The most common complaint was reflux, and a few reported abdominal pain and/or constipation (Supplementary Tables S1 and S2). Only one individual in this study has a clinical diagnosis of ulcerative colitis; this was the NT control whose microbiota showed exacerbated inflammation when transplanted into mice. In general, our data show that the microbiota harbored by NT individuals did not exacerbate intestinal outcomes to the same degree as the microbiota from ASD individuals, suggesting that some individuals with ASD might harbor a microbiota that potentiates GI distress.

More recent work in the microbiota field now supports the idea that many gut diseases, such as IBD and obesity, can result from the loss of beneficial organisms as opposed to the acquisition of pathogenic ones^40^. Based on this, we sought to identify organisms that were reduced in animals that experienced worsened disease. We performed this analysis in two ways: first, by combining all microbiotas together and stratifying based on disease severity; and second, via a more personalized analysis of a single household. These two methods identified two organisms, *B. uniformis* and *Blautia* sp., that were positively correlated with less weight loss and/or colon length. These two organisms have not been mechanistically studied in the context of ASD; however, they have been noted in several observational studies of the microbiota in ASD. In mouse models of ASD, *Blautia* species are reduced in animals genetically predisposed to ASD^18^. In humans with ASD, *Blautia* species are continually demonstrated to be depleted in ASD microbiotas and enriched in NT individuals^35^. In addition, *Blautia* species, specifically, have been found to be depleted in individuals with both ASD and GI complaints^35^. *B. uniformis*, however, has been found to be enriched in both NT and ASD individuals depending on the study and, thus, is not clearly connected with ASD. Despite this, treatment with *B. uniformis* could resolve the behavioral abnormalities observed in mice possessing haploinsufficiency of the chromodomain helicase DNA binding protein (CHD8), one of the top genes associated with ASD^41^. Parameters of GI health were not measured in this study, so it remained unclear whether *B. uniformis* modified local intestinal manifestations.

Our data demonstrate that both *Blautia-*NT and *B. uniformis* can ameliorate pathology and weight loss associated with gut injury induced by DSS. In fact, the worsened colitis severity in mice colonized with the microbiota from one ASD individual could be resolved by oral gavage with *Blautia-*NT, highlighting that individuals with neuro-developmental disorders might lack specific organisms that promote GI health. Moreover, we show that *Blautia-*NT significantly improved the deficit in active social engagement associated with intermittent GI insult. *B. uniformis* treatment also somewhat improved GI insult-associated social behavior abnormalities. A previous study using the maternal immune activation (MIA) model of ASD was able to ameliorate some of the behavioral abnormalities associated with this model using *Bacteroides fragilis* oral treatment^1^. Prior to this study, *B. fragilis* had been shown to improve multiple models of intestinal colitis in mice and induce anti-inflammatory responses within the intestine^42–44^. Thus, these studies and our data collectively highlight that provision of specific members of the microbiota that are lacking in individuals with neurobehavioral abnormalities might improve their GI health and also influence certain aspects of their behavior. This could extend to a variety of diseases whereby GI complications are implicated; for instance, individuals with IBD have been reported to have increased anxiety, depression and asocial behavior. Future studies will be aimed at identifying the mechanism by which *Blautia-*NT is able to ameliorate gut injury-associated behavioral abnormalities.

Collectively, our study suggests that individuals with neuro-developmental diseases might harbor a microbiota that promotes intestinal inflammation, and that this inflammation may also exacerbate specific aspects of their behavioral outcomes. FMT is currently being explored as a therapy for many neuro-developmental diseases; however, the complexity of the community within an FNT makes it difficult to ensure reproducible transplants over time and has the possibility of transplanting unknown pathobionts to the recipient. Thus, identification of specific organisms that can ameliorate GI distress would be useful in this regard. We have identified two such microbes, *Blautia-*NT and *B. uniformis*, that ameliorate GI distress in mice. Future work should further explore the use of these bacteria in other animal models of disease to work toward having specific microbial entities to use in targeted microbiota replacement therapy.

## Methods

### Mice

For all experiments, mice were used in strict adherence to the guidelines set by the University of Utah Institutional Animal Care and Use Committee, as well as with all federal regulations (protocol numbers: 17-04009 and 20-03006). In all, 3-to-4-week-old male C57BL/6J mice were purchased from Jackson Labs and housed/tested in one of 3 mouse facilities at the University of Utah. All animals were housed in a climate-controlled facility with 12-h light/dark cycles, a temperature of 22 °C and 22–30% relative humidity. Mice had ad libitum access to standard mouse chow (Teklad Rodent Diet 2920X, inotiv) and water. Mice were housed in cages with 2–5 mice, and cages were changed every 2 weeks. Germ-free C57BL/6J mice were maintained in gnotobiotic isolators or Tecniplast cages in the germ-free mouse facility at the University of Utah under similar conditions, with autoclaved standard mouse chow.

### DSS-induced intestinal injury model

Overall, 2.5–3.0% DSS (36,000–50,000 MW, Fisher Scientific), depending on mouse facility, was diluted in water and given to mice as the sole source of drinking water to induce intestinal distress. For repeated injury, DSS water was administered to 3–4-week-old mice for 5 days, then mice were given 10 days of regular water, then 5 days of DSS, and finally 10 more days of regular water. Mice were then analyzed at this time point (day 30) or used for behavioral testing. For acute DSS schedule, 6–8-week-old mice were given 2.5% DSS in their drinking water for 6–7 days and assayed daily for change in weight.

### Histological analysis

Whole colons were isolated and fixed overnight in 10% buffered formalin (Fisher Chemical) followed by 70% ethanol. Paraffin-embedded sections were cut longitudinally at 5 μm and stained with hematoxylin and eosin by ARUP laboratories. The entire length of the colon from just under the cecum to the rectum was analyzed by a trained pathologist blind to experimental conditions, and the percentage of crypt loss and percent of colon affected by inflammation was taken into consideration. Therefore, each animal received a score for crypt loss and immune cell aggregation as well as the percentage of the colon affected. For crypt loss severity, a score of 0–3 was given (0 = no crypt loss; 1 = mild crypt loss, most crypts still visible with a few areas effected; 2 = medium severity, greater crypt loss, fewer crypts visible in large areas; 3 = very large areas of total crypt loss, places where crypts are completely gone). For immune cell aggregation: each was given a score from 0–3 that takes into account the level of immune cells present within the lamina propria (0 = no evidence of inflammatory infiltrate; 1 = very low level of cells infiltrating into the tissue; 2 = clear infiltrating lymphocytes into epithelial tissue; 3 = large boluses of inflammatory infiltrates that correspond with areas of crypt loss). The percent of colon affected scoring system was as follows: 0 = no area affected; 0.5 = 1–5%; 1 = 5–20%; 1.5 = 20–30%; 2 = 30–45%; 2.5 = 45–60%; 3: = 60–70%; 3.5 = 70–80%; 4 = > 80%. This was used for both crypt loss and inflammation.

### Behavioral assays

Mice given repeated DSS treatment or mice naive to DSS treatment were allowed 2 h to acclimatize to the behavioral assay laboratory each of the 2 days prior to the beginning of testing. Mice were subjected to a battery of behavioral tests including three-chamber, open field, novel object recognition, elevated plus-maze, tail suspension, pre-pulse inhibition and marble burying tests. In each test, isopropyl alcohol was used to thoroughly clean the in-use apparatus between trials. This was done to remove potential remaining odors that could confound results.

#### Three-chamber test

This test was performed in a three-chamber opaque acrylic box. An empty central compartment connected with two lateral compartments (all 20 × 20 cm). Two cylindrical wire cup enclosures (10 × 6 cm in diameter) were placed in the center of the lateral compartments containing an age- and weight-matched male foreign conspecific (acclimated extensively to the enclosures before testing) or an inanimate object of equal size. The test mouse was acclimated to the empty apparatus and allowed to freely explore for 10 min, 24 h prior to testing. The day after, the mouse was placed in the central compartment and allowed to explore the social and non-social compartments for 10 min. The placement of the wire cup enclosures was counterbalanced across treatments. Behavioral measures include the number of social approaches and the duration of social investigation/interaction. Transitions between compartments and active sniffing behavior directed toward counterpart/object were hand-scored offline. Social/object contacts were defined as active sniffing, pawing, and touching of the experimental subject in proximity (<1 cm) to the edge of the wire cup enclosure.

#### Open-field test

Animals were placed in the center of a black Plexiglass open-field arena (40 × 40 × 40 cm) and allowed to explore freely for 5 min. Horizontal locomotor activity and spontaneous behaviors were scored and analyzed using behavioral tracking software (EthoVision XT).

#### Novel object recognition test

Mice were individually acclimatized to Makrolon cages for 15 min each. The day after, animals were exposed to two unfamiliar black plastic cylinders (8 cm tall × 3.5 cm in diameter) affixed to the floor and symmetrically placed at 6 cm from the two nearest walls. Mice were placed in a corner, facing the center and at equal distance from the two objects. Their start position was rotated and counterbalanced for each treatment throughout the test. Twenty-four hours later, mice were placed in the same cage for memory testing. One of the cylinders was replaced by an unfamiliar plastic rectangular block (6 cm tall × 3 × 3 cm), which was placed in a counterbalanced fashion to avoid experimental bias. Both sessions were videotaped for 15 min, and hand-scored by a reviewer at a later date. The number and total duration of exploratory approaches between the unfamiliar and familiar objects were measured. Exploration was defined as sniffing or touching either of the two objects with the snout; sitting on the object was not considered exploration. An object exploration index was calculated as the ratio of the duration of the exploratory approaches targeting the unfamiliar objects over the time of exploration of both objects.

#### Elevated plus-maze test

A black Plexiglass apparatus with a light gray floor and two open arms (25 × 5 cm) and two closed arms (25 × 5 × 5 cm) that Supplementary from a central platform (5 × 5 cm) at 60 cm from the ground was utilized. Mice were placed individually in the center square facing an open arm and allowed to explore the maze for 5 min. Behavioral analysis was performed by an observer blinded to experimental condition and included the following parameters: time spent in open and closed arms and in the central platform; number of open and closed arm entries; total entries; stretch-attend postures; head dips. An arm entry was counted only when all four paws were inside the arm.

#### Tail suspension test

Mice were individually suspended by the tail using medical tape affixed to a hook 30 cm above the floor. Environmental light was kept at 300 lux. Animals were video recorded for 6 min, and the duration of immobility, the latency to immobility, and number of fecal boli were measured.

#### Marble burying test

Mice were allowed to habituate to a sawdust filled cage for 10 min. At the end of this phase, the mouse was briefly removed, and 24 glass marbles were placed on the surface of the sawdust in the cage at even distances. The animal was then reintroduced to the cage, and its behavior was monitored for 10 min. The number of digging bouts, the overall digging duration and the number of buried marbles were scored. A marble was considered buried if at least two-thirds of its surface area was covered in sawdust.

### Colonization with human microbiotas

Participants gave written informed consent for data and sample collection and use under IRB_00006042, Genetics of Autism. Families were selected that contained at least one individual diagnosed with ASD and gastrointestinal complaint. Information regarding donor health, disease, and medication usage was collected (Supplementary Tables S1 and S2). Fecal samples were collected and dated by donors and stored in their freezers until pickup. After collection, bulk fecal samples were stored at −80 °C until processing and colonization into mice. Mice were colonized following the protocol in Goodman et al., description following^45^. Fecal samples were allowed to thaw at room temperature and suspended in reduced PBS at 15 g/mL in 15-mL conical tubes. Samples were vortexed for 5 min and then rested for 5 min to allow solid particles to precipitate to the bottom of the tubes. In all, 200 µL aliquots of the suspended liquid fractions were then gavaged into germ-free mice: mice were removed from gnotobiotic isolators, immediately inoculated with human microbiotas and then housed in secured Tecniplast Iso-cages with HEPA filters, segregated by microbiota sample. C57BL/6J mice were utilized for their availability as GF mice, as well as their tractability for potential, future genetic manipulation.

### 16S rRNA gene sequencing

Fecal input samples and large intestinal luminal contents were collected for 16S sequencing of bacterial communities. DNA was isolated from contents using a Zymo Quick-DNA Fecal/Soil Microbe 96 Magbead kit according to the manufacturer’s instructions. Fluorometric measurement (Qubit) was used to quantify and then normalize DNA concentrations used as templates in PCR. A single round of PCRs was performed in triplicate using custom 16 S targeting primers (below) on the 3’ end of long oligonucleotides, including the 8 nucleotide sample-specific barcode and Illumina adapter sequences described in ref. ^46^. Two 16S sequencing experiments were included in this work using different 16S rRNA gene targeting sequences. Both amplify V3 and V4 16 S regions and used the same set of barcodes but employed different 16 S rRNA gene targeting primer sequences. One run included fecal samples from colonized mice and all familial donors and utilized the 16 S targeting sequences with 2 nucleotide pad (underlined) 5’-TGCCTACGGGNBGCASCAG-3’ and 5’-GCGACTACNVGGGTATCTAATCC-3’ (Fig. 3a–f and Supplementary Fig. 3). The other run only included family 3 donors and mice colonized with family 3 and utilized the 16 S targeting sequences with 2 nucleotide pad (underlined): 5’-TAGGGRGGCWGCAGTRRGG-3’ and 5’-TTCTACHVGGGTATCTAATCCTGTT-3’ (Fig. 3g). Gel electrophoresis was utilized to determine the successfulness of PCRs, triplicates combined and PCR cleanup was performed using Axygen Magprep PCR cleanup kit according to the manufacturer’s instructions. PCR products were then quantified using QuantIT picogreen (Invitrogen) and these final sequence libraries were evenly multiplexed. Samples were submitted to the Genomics Core at the University of Utah. 5% PhiX was included and samples were sequenced on Illumina Miseq with paired-end 300 cycle sequencing.

### Analysis of 16S data

Analysis of 16S data was performed with QIIME and QIIME2 utilizing the Center for High-Performance Computing at the University of Utah. Cutdapt trim-pairs was used to remove primers from demultiplexed sequences. Vsearch join-pairs were used to join paired-end sequencing reads. Quality-filter q-score-joined was used to remove low-quality reads (with a minimum PHRED score of 10). Deblur denoise-16S was used for quality control. Vsearch uchime-denovo was used to filter chimeras. Rooted Phylogenetic tree was assembled using phylogeny align-to-tree-mafft-fasttree pipeline. Samples were then rarefied to 10,000 reads by using feature-table rarify. To determine beta-diversity metrics between microbiota samples, the diversity core-metrics-phylogenetic pipeline was used. Unweighted UniFrac distances were used to compared beta diversity between samples. To determine if individual taxa were differentially abundant between samples, we utilized the composition ANCOM (Analysis of Composition of Microbiota). Before ANCOM, taxa were collapsed, and pseudocounts added to allow for analysis at different taxonomic levels.

### Culture collection and *Blautia* isolation

Feces collected from mice colonized with microbiota 1306012 were collected and used to assemble a culture collection as described in ref. ^45^. Feces were plated on Gut Microbiota Medium (GMM) in an anaerobic chamber and allowed to incubate for 24 h at 37 °C. Colonies were harvested from the plates and frozen for further use. Frozen stocks were diluted to determine the cell concentration. Using this calculation, cells were diluted into 96 well plates at the proper concentration to maximize the number of wells that contain 1 bacterial cell. These plates were allowed a week for growth to occur. Turbid wells were then subject to 16S sequencing and preserved in glycerol at −80 °C.

### Treatment with bacterial supplements

Frozen stocks of either *Blautia-*NT (referred to as isolate JLR.GB024) or *Bacteroides uniformis* (ATCC) were thawed, spun down at 5000× *g* for 5 min, and resuspended in reduced PBS. Mice were then gavaged with 10^7^ CFU of bacteria beginning two weeks before DSS treatment and during the DSS treatment and water treatment phases. For the acute DSS time course, mice were gavaged daily, and for the 30-day model, mice were gavaged every 2–3 days.

### Statistics and reproducibility

Mouse sample sizes were chosen based on either those utilized in similar studies and/or the availability of mice. The latter is true for experiments that utilized age-matched offspring of breeder pairs colonized with human microbiotas. In these experiments, all male offspring in each age-matched litter were utilized. Follow-up behavioral study sample sizes were estimated using the calculated Eta square from the original set of results. Randomization was utilized throughout this work, specifically in selecting GF mice for colonization with human microbiotas, in selecting which mice were to receive which treatments in colitis experiments, and the order in which mice were behaviorally tested. Blinding was utilized in both scoring behavioral testing and histological sections. Due to the higher prevalence of ASD in male humans and better susceptibility to DSS than females, male mice were selected for analysis. No data were excluded from the analyses. Generally, experiments were repeated twice and were reproducible: colitis social deficits were observed multiple times, and rescue of disease with bacterial treatment was also replicated in multiple experiments.

### Reporting summary

Further information on research design is available in the Nature Portfolio Reporting Summary linked to this article.

### Supplementary information

Supplementary Information

Peer Review File

Reporting Summary

### Source data

Source Data

## Data Availability

*The Blautia sp*. JLR.GB0024 genome assembly and raw reads have been deposited in the NCBI database under accession number PRJNA952791. The 16 S rRNA gene sequence data and associated metadata is available under NCBI BioProject accession number PRJNA952791. There are no restrictions on access to this data. Source data are provided with this paper.

## Code availability

No novel code was generated for this work.
