# Supplementary material for: Colitis reduces active social engagement in mice and is ameliorated by supplementation with human microbiota members
Source: Nat Commun. 2024 Mar 30;15:2769. doi: 10.1038/s41467-024-46733-7 (PMC10980768; doi:10.1038/s41467-024-46733-7)
Supplement: Supplementary file 1 — Supplementary Information [file 41467_2024_46733_MOESM1_ESM.pdf]

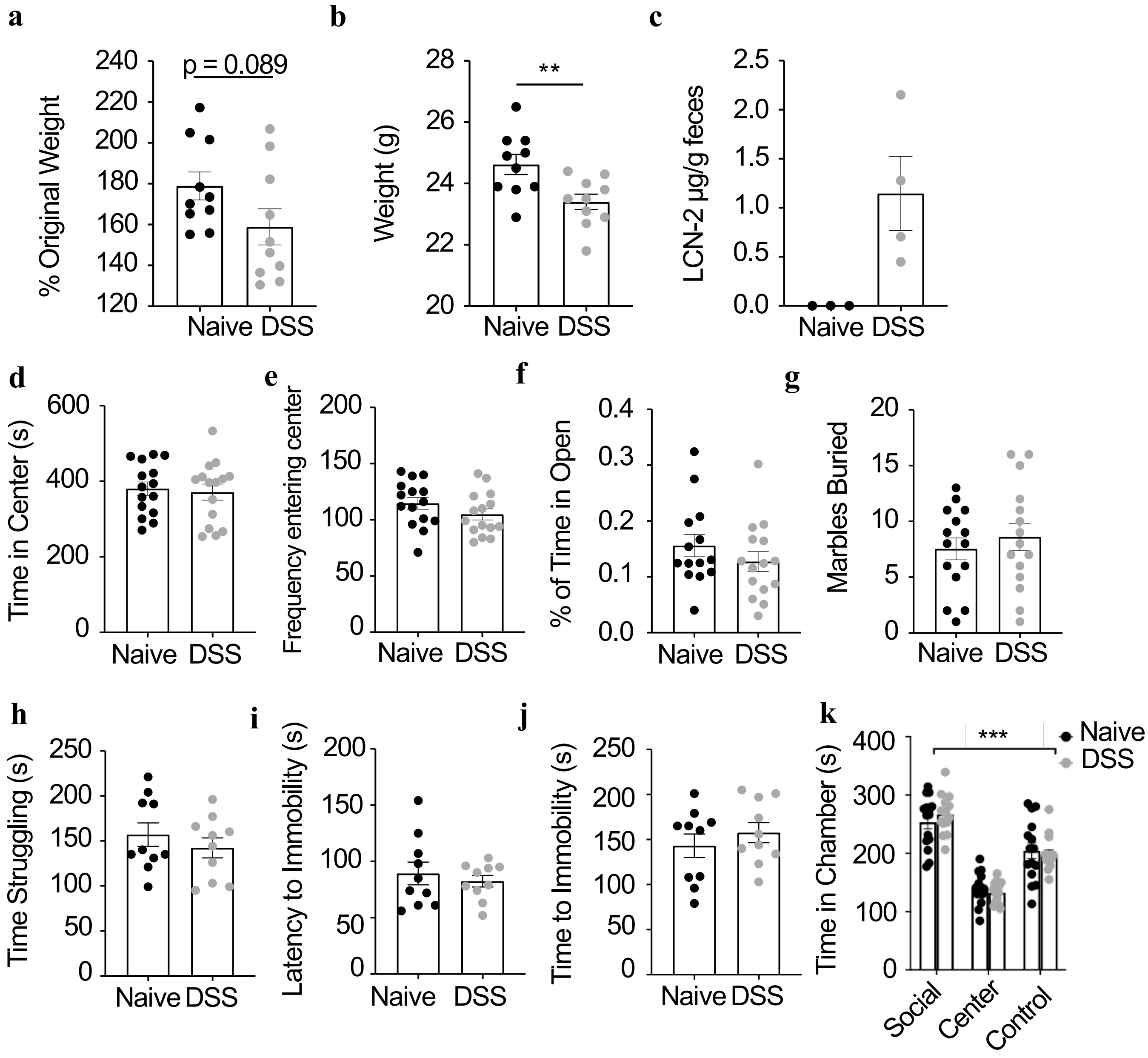

Supplementary Figure 1. **Behavioral phenotypes not affected by repeated DSS treatment.**

Black circles represent naïve mice, and gray circles represent DSS-treated mice. (**a-c**) Mice given repeated DSS were analyzed for colitis severity after behavioral analysis (n=10 animals/treatment). (**a**) Percent of original weight (p=0.0893), (**b**) total weight (p=0.0086), and (**c**) fecal lipocalin (n=3-4 animals/treatment) were measured. Mice were assayed using an open field test (n=15 animals/treatment) (**d-f**). During analysis, the arena was divided into center and peripheral areas. The total time (**d**) entering the center and frequency of times (**e**) entering the center were analyzed. Mice were assayed using the elevated plus maze and (**f**) assayed for total time on the open arms of the maze, divided by total time tested. (**g**) The marble burying test was conducted, and the number of marbles buried was assayed (n=15 animals/treatment). (**h-j**) The tail-suspension test was conducted (n=10 animals/treatment), and the time spent struggling (**h**), latency to first stop (**i**), and total time suspended, not struggling (**j**) was analyzed. (**k**) Time spent within each chamber, during 3-chamber test (n = 15 animals/treatment). Statistics: All bars represent mean values +/- SEM. ** $P < 0.01$ *** $P < 0.001$ for unpaired, two tailed T test or two way ANOVA **(k).**

a

b

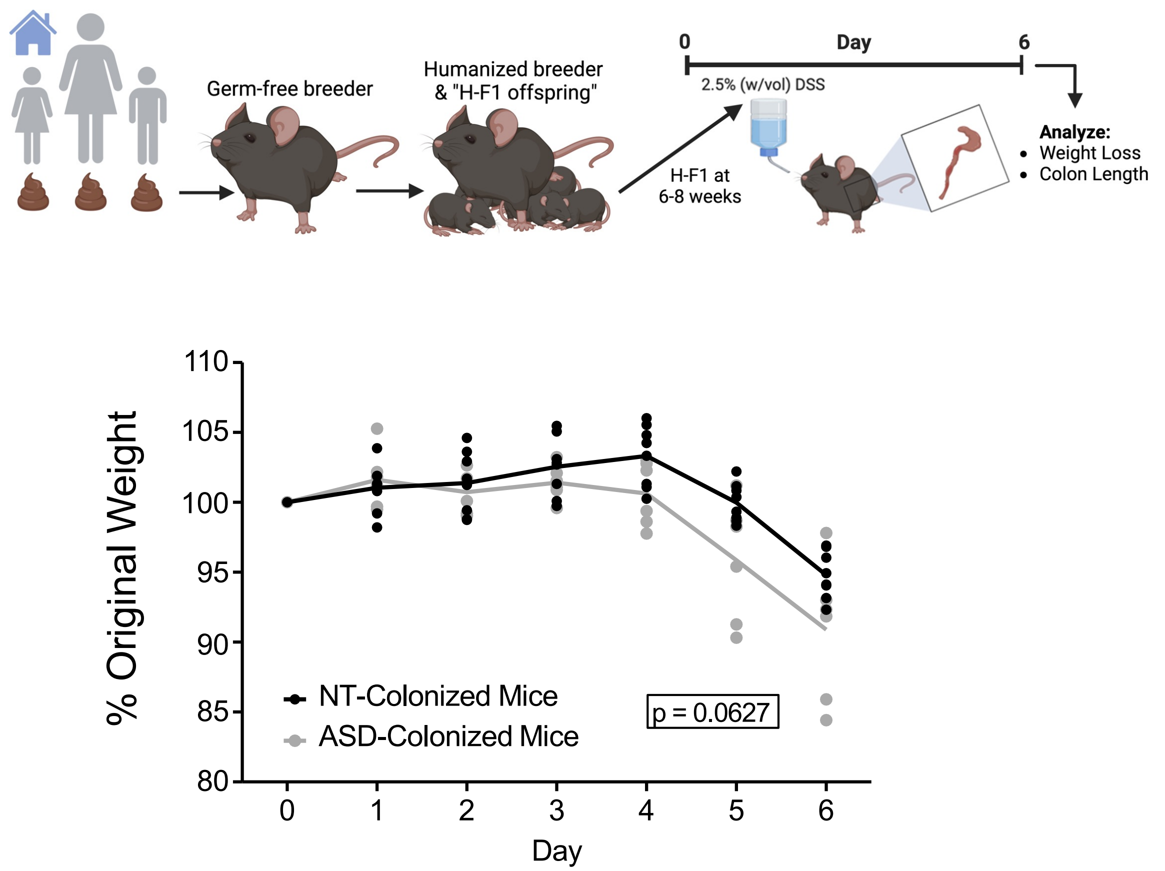

Supplementary Figure 2**. Percent of original weight by neurotype.**

(a) Diagram of experimental design. **(b)** Acute DSS colitis: the percent original weight of all mice from an individual microbiota were averaged (displayed as circles, n=6,8 animals/treatment). The means of these averages were calculated when grouped by neurotype (plotted as the line). Gray represents ASD-associated microbiotas; black represents NT-associated microbiotas. Statistics: Lines represent mean values +/- SEM. p=0.0627 via repeated measures 2-way ANOVA.

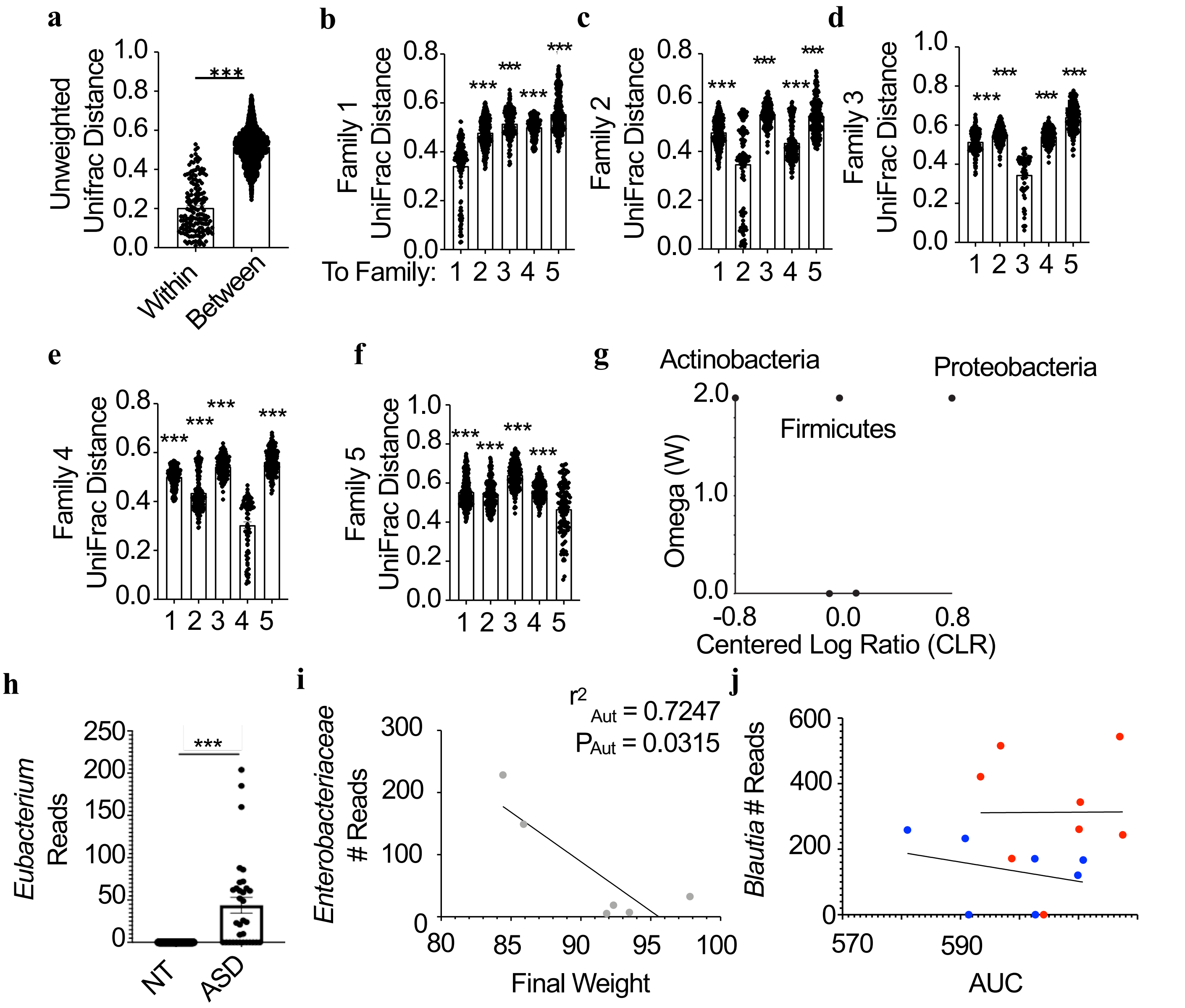

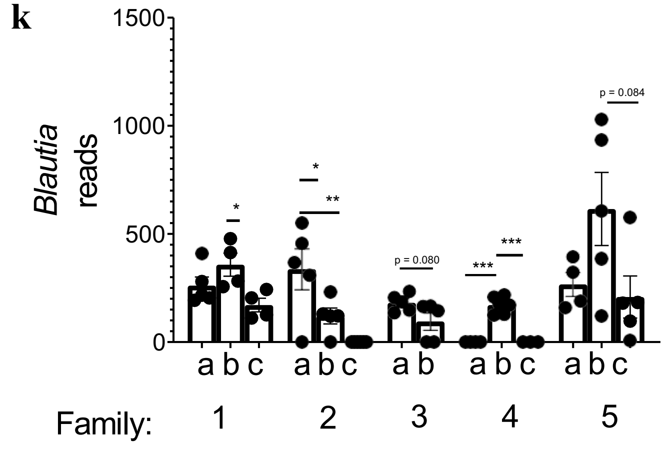

Supplementary F igure 3. **Characterization of microbiotas.** (**a**) Unweighted UniFrac distance values, comparing within vs between sample (n = 161, 5378 analyses). (**b-f**). Unweighted Unifrac distances when making within-family and between family comparisons (n=66-304 analyses). (**g**) ANCOM analysis at the phylum level comparing donor neurotype. (**h**) *Eubacterium sp.* reads by donor neurotype. (**i**) Regression of *Enterobacteriaceae sp.* with weight at the end of six days of DSS. (**j**) Regression of *Blautia sp.* with AUC during acute DSS. Red is NT and blue is ASD. (**k**) *Blautia*-NT reads in mice, by family and donor. Statistics: All bars represent mean values +/- SEM. * $P < 0.05$ ** $P < 0.01$ *** $P < 0.001$ two-tailed unpaired student's T-test (**a,h**) or one-way ANOVA with Tukey's multiple comparisons (**b-f,k**).

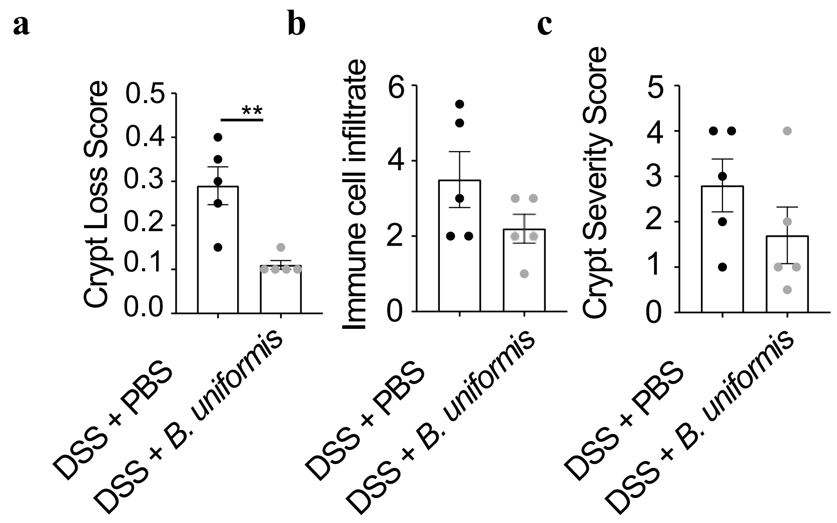

Supplementary Figure 4. **Extended details regarding histology scoring in mice.** Mice did (black) or did not (gray) receive *B. uniformis* treatment (n=5 animals/treatment). **(a)** Crypt loss**, (b**) immune cell infiltrate, and **(c)** crypt severity scoring associated with total histology scores. Statistics: All bars represent mean values +/- SEM. ** $P < 0.01$ two-tailed unpaired student's T-test.

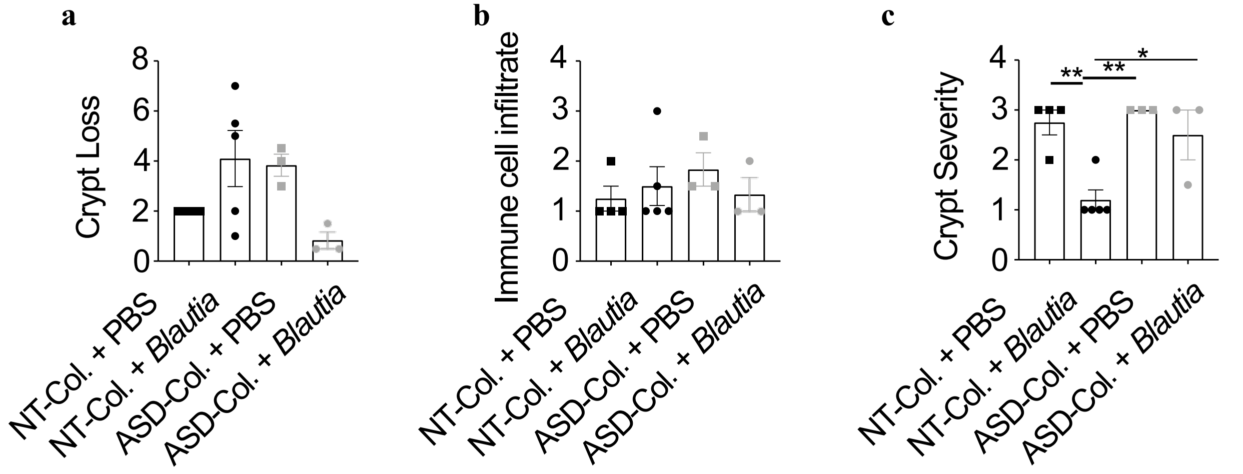

Supplementary Figure 5. **Extended details regarding histology scoring in mice.**

Mice were colonized (Col.) with either microbiota (NT = black, ASD = gray) and treated with *Blautia*–NT (circles) or with vehicle control (PBS, squares) (n=3-5 animals/treatment). **(a)** Crypt loss**,** (**b**) inflammation, and **(c)** crypt severity scoring associated with total histology scores. Statistics: All bars represent mean values +/- SEM. * $P < 0.05$, ** $P < 0.01$ ordinary one-way ANOVA with Tukey's multiple comparisons test.

Table S1: Donor Diagnosis and Key.

Family	Donor ID (in Fig 2)	Diagnosis	Donor	Gender	Birth Year
1	a	Typically Developing	Mother	F	1961
1	b	Typically Developing	Sibling	M	1990
1	c	Autism	ASD	M	1988
1	Excluded	Language delay	Child	F	2015
2	a	Autism	Father	M	1988
2	b	Autism	ASD	M	1997
2	c	Typically Developing	ASD	M	1962
3	a	Typically Developing	Mother	F	1962
3	b	Autism	ASD	M	1995
4	a	Typically Developing	Mother	F	1977
4	b	Typically Developing	Sibling	M	2005
4	c	Autism	ASD	M	2001
5	a	Typically Developing	Mother	F	1944
5	b	Typically Developing	Sibling	F	1967
5	c	Autism	ASD	F	1965

Table S2: Donor Diagnosis and Medical History.

Paper ID #	Birth Year	Gender	GI Comorbidies	Medical	Medications	Diagnosis
1a	1961	F	Constipation; Gastroesophageal reflux disease; Ulcerative Colitis; Lactose Intolerance; chronic diarrhea, blood bowl movements, abdominal pain/cramps; Hemorrhoids	Seasonal Allergies; Palpitations; sarcoidosis; Arthritis; Stiffness; easily fatigued; Imbalance/Coordination; paraesophageal hernia surgery, Gallbladder hernia surgery, nissen fundopliation	Prozac; Aspirin; Lyrica; Percocet; Remicade; Senna; Prevacid; Meloxicam; Plaquenil	NT
1b	1990	M	Constipation - evaluated by gasteroenterologist; Abdominal pain/cramps	Seasonal Allergies; asthma	Allegra D; Omeprazole	NT
1c	1988	M	Gastreoesophageal reflux disease;		Prozac, Prilosec	ASD
2a	1962	M	IBS; Ulcerative Colitis;	High Cholesterol; Hearing loss; Ringing in the ears; Seasonal Allergies; wears glasses	Ibuprofen; Multi-Vit; Aspirin; Calcium Gummy	NT
2b	1997	M	Lactose Intolerance; Diert: no processed sugars	Ringing in the ears; Seasonal Allergies	Flonase; Qvar	ASD
2c	1988	M	Appendectomy 2000; IBS; Loose stool	Seasonal Allergies;	None	ASD
3a	1962	F	None	Irregular heart rhythm; Heart murmur; Seasonal Allergies; Cataracts; Fractured Bones; Pain with chewing; Anemia; Thyroid Disease; Hernia repair 1972; allergy symptoms within the past month	Levothyroxine; Metoprolol; Simvastatin; Multi-Vit; Vit-B Complex; Vit-D; Ibuprofen; Marine Minerals Electrolyte Energy Recharger; chlortab allergy	NT
3b	1995	M	Constipation; Encopresis	Seasonal Allergies; Asthma; Tremor; Bed wettting; Constipation; Encopresis; Within the past month-Seasonal allergies, fever, aches, stuffy nose, congestion	Guanfacine; Melatonic; Abilify; Sesame Seed Oil; Fiber Gummies; Chlovtyab Allergy; Various Doterra Oils; Total Mercury - homeopathic	ASD
4a	1977	F	Constipation	Irregular heart rhythm; high blood pressure; Frequent Headaches; Insomnia; Anemia; Tuberculosis; UTI; Hemorrhoids; Galbladder removed 2003; Liver Biopsy 2008 (inflammed liver); Borken arm surgery 1982; cold last week with sore throat, runny nose, cough	Vit D, Vit B12; Multi Vit; Probiotic; Lexapro	NT
4b	2005	M	none	Seasonal allergies; Asthma; Hx. Fractured bone	Singular; Vit D; Zyrtec; Symbcort	NT
4c	2001	M	Constipation; Gastroesophageal reflux disease; Encopresis	Seasonal Allergies; Tooth Decay; Insomnia; Obstructive sleep apnea (tonsillectomy addenoidectomy 2004);	Depakote; Resperidone; Clonidine; Kapray; Abilify; Mirilox; N-Acetyl Cysteine Supplement	ASD
5a	1944	F	Gastroesophageal reflux disease;	Hx Heart Attack; Irregular Heart Rhythm; Palpitations; Chest Pain; Frequent Headache; Cataracts; Obstructive sleep apnea; Arthritis/Rheumatism; tooth decay; Chronic Rash; Hepatitis; UTI Hx.; Food Intolerance (gluten/casein); Appendectomy 1961; Hysterectomy 1990; Gallbladder removed 1999; Total R-Knee 2013; Total L-Knee 2017; Cataracts removed bilaterally.	Omeprazole; Amiodarone; Sumatriptan; Warfarin; Xaralto; Tramadol; Miralax; Klonopin	NT
5b	1967	F	Food Intolerances (gluten/casein, no processed sugars) Last April I did a juicing/fasting body cleanse. Since that time my body only comfortably tolerates food on a mucusless diet - fritus and non-starchy vegetables; Abdominal pain/cramps; Difficulty swallowing; Constipation	Epstein Barr Virus; Deviated septum 1995; Tonsillectomy 1999; Benign cyst L-armpit removed 2003; L-Hip labral repair & femoroacetabular impingement surgery 2013; Heart Murmer; Chest Pain; Ringing in ears; Frequent Headache; Vision loss; Asthma; Arthritis/Rheumatism; Fractured bone; fatigue easily; Pain or tender scalp; toot decay; Anemia; Unusual susceptibility to infection;	None	NT
5c	1965	F	Gluten free / Casein Free diet; Constipation; Abdominal Pain/Cramps	Obstructive sleep apnea; Seasonal Allergies; Tooth Decay; Fractured bone;	Calcium; Magnesium; Zinc; Vit- D	ASD
