# Supplementary material for: Colitis reduces active social engagement in mice and is ameliorated by supplementation with human microbiota members
Source: Nat Commun. 2024 Mar 30;15:2769. doi: 10.1038/s41467-024-46733-7 (PMC10980768; doi:10.1038/s41467-024-46733-7)
Supplement: Supplementary file 3 — Reporting Summary [file 41467_2024_46733_MOESM3_ESM.pdf]

nature portfolio

Corresponding author(s): June Round

Last updated by author(s): 1/31/2024

Reporting Summary

Nature Portfolio wishes to improve the reproducibility of the work that we publish. This form provides structure for consistency and transparency in reporting. For further information on Nature Portfolio policies, see our Editorial Policies and the Editorial Policy Checklist.

Statistics

For all statistical analyses, confirm that the following items are present in the figure legend, table legend, main text, or Methods section.

n/a	Confirmed	
☐	☒	The exact sample size (*n*) for each experimental group/condition, given as a discrete number and unit of measurement
☐	☒	A statement on whether measurements were taken from distinct samples or whether the same sample was measured repeatedly
☐	☒	The statistical test(s) used AND whether they are one- or two-sided<br>*Only common tests should be described solely by name; describe more complex techniques in the Methods section.*
☒	☐	A description of all covariates tested
☐	☒	A description of any assumptions or corrections, such as tests of normality and adjustment for multiple comparisons
☐	☒	A full description of the statistical parameters including central tendency (e.g. means) or other basic estimates (e.g. regression coefficient) AND variation (e.g. standard deviation) or associated estimates of uncertainty (e.g. confidence intervals)
☐	☒	For null hypothesis testing, the test statistic (e.g. *F*, *t*, *r*) with confidence intervals, effect sizes, degrees of freedom and *P* value noted<br>*Give P values as exact values whenever suitable.*
☒	☐	For Bayesian analysis, information on the choice of priors and Markov chain Monte Carlo settings
☒	☐	For hierarchical and complex designs, identification of the appropriate level for tests and full reporting of outcomes
☒	☐	Estimates of effect sizes (e.g. Cohen's *d*, Pearson's *r*), indicating how they were calculated

Our web collection on statistics for biologists contains articles on many of the points above.

Software and code

Policy information about availability of computer code

Data collection	*Provide a description of all commercial, open source and custom code used to collect the data in this study, specifying the version used OR state that no software was used.*
Data analysis	QIIME and QIIME2 were used to analyze 16s rRNA data in this study.

For manuscripts utilizing custom algorithms or software that are central to the research but not yet described in published literature, software must be made available to editors and reviewers. We strongly encourage code deposition in a community repository (e.g. GitHub). See the Nature Portfolio guidelines for submitting code & software for further information.

Data

Policy information about availability of data

All manuscripts must include a data availability statement. This statement should provide the following information, where applicable:

- Accession codes, unique identifiers, or web links for publicly available datasets
- A description of any restrictions on data availability
- For clinical datasets or third party data, please ensure that the statement adheres to our policy

All sequencing reads and associated data are available under NCBI BioProject PRJNA952791.

Human research participants

Policy information about studies involving human research participants and Sex and Gender in Research.

Reporting on sex and gender	Samples from human subjects were taken from both sexes. Donor details, including sex, can be found in supplemental table 1 and figure 2. As familial controls were utilized, and limited subjects were available, sex-based controls/analyses were not utilized.
Population characteristics	These data can be found in supplemental table 1 and 2. All families included contained at least one individual with autism and GI complaint. Information regarding IBD diagnosis is also provided. Reported medical issues and medicines taken are also utilized.
Recruitment	Samples were collected from families in the Utah Population Database. Families were selected with at least one individual with ASD and GI complaint. Racial and ethnic underrepresentation may be present due to the demographics of Utah and the small number of families included.
Ethics oversight	The institutional animal care and use committee at the University of Utah

Note that full information on the approval of the study protocol must also be provided in the manuscript.

Field-specific reporting

Please select the one below that is the best fit for your research. If you are not sure, read the appropriate sections before making your selection.

[x] Life sciences [ ] Behavioural & social sciences [ ] Ecological, evolutionary & environmental sciences

For a reference copy of the document with all sections, see nature.com/documents/nr-reporting-summary-flat.pdf

Life sciences study design

All studies must disclose on these points even when the disclosure is negative.

Sample size	For most experiments with SPF mice, sample size was determined by following sample sizes used in similar experiments/publications. Follow-up behavioral sample sizes were estimated using the calculated eta square from the original set of results. For experiments with germ-free mice, sample sizes were based on availability of age-matched, male mice. For figure 2, sample size was not pre-calculated. Instead, all age-matched male mice from breeders colonized with an individual family's microbiomes were used.
Data exclusions	No data were excluded from analysis.
Replication	Colitis social deficits were observed multiple times. Rescue of disease with bacterial treatment was also replicated in multiple experiments.
Randomization	All mice were randomly assigned to each group before each experiment.
Blinding	Histological analyses and scoring of behavioral assays were performed blinded.

Reporting for specific materials, systems and methods

We require information from authors about some types of materials, experimental systems and methods used in many studies. Here, indicate whether each material, system or method listed is relevant to your study. If you are not sure if a list item applies to your research, read the appropriate section before selecting a response.

Materials & experimental systems

n/a	Involved in the study	
[x]	[ ]	Antibodies
[x]	[ ]	Eukaryotic cell lines
[x]	[ ]	Palaeontology and archaeology
[ ]	[x]	Animals and other organisms
[x]	[ ]	Clinical data
[x]	[ ]	Dual use research of concern

Methods

n/a	Involved in the study	
[x]	[ ]	ChIP-seq
[x]	[ ]	Flow cytometry
[x]	[ ]	MRI-based neuroimaging

Animals and other research organisms

Policy information about studies involving animals; ARRIVE guidelines recommended for reporting animal research, and Sex and Gender in Research

Laboratory animals	Male C57Bl6 mice between 3-6 weeks of age were utilized in these studies.
Wild animals	The study did not involve wild animals.
Reporting on sex	All mice utilized were male mice. This was chosen as ASD is sex-skewed towards males.
Field-collected samples	NA.
Ethics oversight	All work detailed in this manuscript was performed in strict accordance with the recommendations in the Guide for the Care and Use of Laboratory Animals of the National Institutes of Health. All of the animals were handled according to approved institutional animal care and use committee (IACUC) protocol # 17-04009 at the University of Utah.

Note that full information on the approval of the study protocol must also be provided in the manuscript.
