# Supplementary material for: Colitis reduces active social engagement in mice and is ameliorated by supplementation with human microbiota members
Source: Nat Commun. 2024 Mar 30;15:2769. doi: 10.1038/s41467-024-46733-7 (PMC10980768; doi:10.1038/s41467-024-46733-7)
Supplement: Supplementary file 2 — Peer Review File [file 41467_2024_46733_MOESM2_ESM.pdf]

nature portfolio

Peer Review File

Colitis reduces active social engagement in mice and is ameliorated by supplementation with human microbiota members

Open Access This file is licensed under a Creative Commons Attribution 4.0 International License, which permits use, sharing, adaptation, distribution and reproduction in any medium or format, as long as you give appropriate credit to the original author(s) and the source, provide a link to the Creative Commons license, and indicate if changes were made. In the cases where the authors are anonymous, such as is the case for the reports of anonymous peer reviewers, author attribution should be to 'Anonymous Referee' followed by a clear attribution to the source work. The images or other third party material in this file are included in the article's Creative Commons license, unless indicated otherwise in a credit line to the material. If material is not included in the article's Creative Commons license and your intended use is not permitted by statutory regulation or exceeds the permitted use, you will need to obtain permission directly from the copyright holder. To view a copy of this license, visit http://creativecommons.org/licenses/by/4.0/.

Editorial Note: This manuscript has been previously reviewed at another journal that is not operating a transparent peer review scheme. This document only contains reviewer comments and rebuttal letters for versions considered at *Nature Communications.*

REVIEWER COMMENTS

Reviewer #1 (Remarks to the Author):

The authors have reasonably responded to comments from prior peer review cycle. They should include in limitations that one of the humanizations was from an ulcerative colitis donor.

We thank the editors and reviewers for their comments and have made the suggested changes. You will see within the manuscript the requested textual changes in yellow highlight. These changes are described below in detail in bold.

Reviewer #1 (Remarks to the Author):

The authors have reasonably responded to comments from prior peer review cycle. They should include in limitations that one of the humanizations was from an ulcerative colitis donor.

We have now included that one of the humunizations was from a UC donor. This can be found in the second paragraph in the discussion and is highlighted in yellow.
This is copied and pasted below from the manuscript.

"Only one individual in this study had a clinical diagnosis of inflammatory bowel disease (IBD), ulcerative colitis, and this was one of the NT controls, whose microbiota showed exacerbated inflammation when transplanted into the mouse."
